# Cervical Spine Involvement in Morphea Patients With Subclinical Neurologic Signs

**DOI:** 10.7759/cureus.104925

**Published:** 2026-03-09

**Authors:** Michael Critelli, Taylor Davis, Hayden Flume, Yousef Sarameh, Clayton B Baer, Roban Shabbir, John T Schwartz

**Affiliations:** 1 Texas College of Osteopathic Medicine, University of North Texas Health Science Center, Fort Worth, USA; 2 Arizona College of Osteopathic Medicine, Midwestern University, Glendale, USA; 3 School of Medicine, Mercer University, Macon, USA; 4 Lewis Katz School of Medicine, Temple University, Philadelphia, USA; 5 Orthopedic Surgery, Valley Consortium for Medical Education, Modesto, USA

**Keywords:** cervical spine deformity, linear scleroderma, morphea, neurologic signs, orthopedic spine surgery, radiologic findings

## Abstract

Morphea is a rare skin disease that involves the fibrosis of subcutaneous tissues. Several subtypes of morphea, such as linear and deep variants, may affect musculoskeletal structures and produce neurologic manifestations. However, morphea is differentiated from systemic scleroderma by the absence of internal organ damage. Although the prognosis of a morphea diagnosis is generally favorable, systemic involvement can be severe. However, early detection may improve disease management. The relationship between morphea and its involvement in the cervical spine remains incompletely characterized. As such, this narrative review examines cervical spine involvement in patients with morphea. OpenEvidence, PubMed, and Google Scholar databases were used to identify relevant articles. MeSH terms included “scleroderma, localized”, “cervical vertebrae”, and “neurologic manifestations”. Keywords searched included “morphea”, “cervical”, “neurologic”, and “subclinical”. The search was restricted to English-language articles written in the last 10 years. In addition to the database searches, independent reviewers performed a thorough citation search and included studies relevant to the topic. The information in the articles was assessed to determine relevant signs and symptoms in patients with morphea that had cervical spine involvement. Almost half of all head-and-neck morphea patients presented with an abnormal MRI finding. Some patients presented with incidental findings, such as white matter lesions or vascular malformations, and later developed clinical symptoms, including seizures or migraines. Additionally, the hardening of subcutaneous tissues in the cervical spine can obstruct the neurovasculature, ranging from subtle to significant. Fleeting symptoms such as recurrent numbness or tingling can indicate invasive tissue growth. Although the disease is generally self-limiting, surgery is indicated in cases of contracture, while some patients may opt for cosmetic operations. Although morphea starts as a disease of the skin, it can advance to affect the underlying tissues. Several cases have documented the dangerous effects of the disease when infiltrative tissue growth affects organ systems beneath the skin. Periodic imaging studies, careful physical exam, and detailed history taking aid in the early diagnosis of the disease. For this reason, it is necessary to follow up with morphea patients regularly to observe the progression of the disease.

## Introduction and background

Morphea, or localized scleroderma, is a chronic autoimmune and inflammatory condition in which excess collagen accumulates in the skin and, in some cases, subcutaneous fat, fascia, muscles, or bone leading to sclerosis of the affected tissue [1,2]. Currently, the etiology of morphea is not fully understood; however, it is believed that both genetic and environmental factors play a role [3]. Morphea is a rare disease, with a prevalence of about 0.4 to 2.7 per 100,000 people [2]. Females are affected more than males, and pediatric morphea accounts for about half of all cases, with a higher prevalence observed in White populations [2,4]. Although rare, morphea is a disease that can have debilitating effects when morphea implications arise in the cervical spine. A focused review of cervical spine involvement is warranted because deep fibrotic extension in the cervical region may affect neural structures and spinal biomechanics even in the absence of overt systemic disease. Therefore, subclinical cervical spine involvement may carry meaningful neurologic implications.

**Figure 1:**
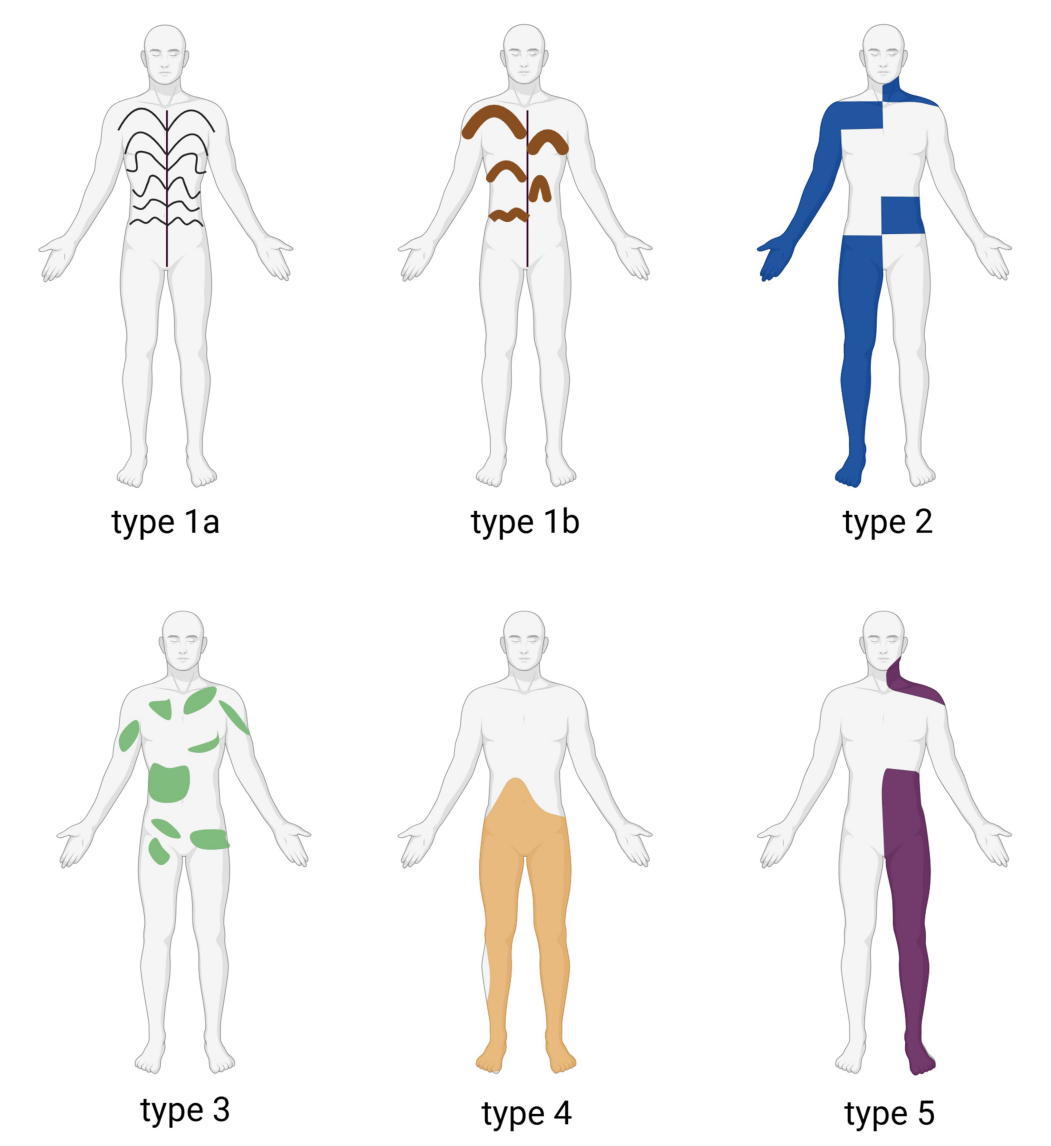
Distribution types of linear scleroderma Type 1a. Following the Blaschko line, narrow sclerotic plaques. Type 1b. Following the Blaschko line, broad sclerotic plaques. Type 2. Grid pattern. Type 3. Leaf-like pattern. Type 4. Patches pattern bilaterally. Type 5. Lateralized pattern. This image is borrowed from Takahashi et al. [10] and is republished with permission obtained from Multidisciplinary Digital Publishing Institute (MDPI).

Morphea usually progresses in stages. It starts with an inflammatory phase with erythematous plaques, warmth, and swelling. It then progresses into a sclerotic phase, followed by atrophy of the affected tissues [2,5]. Most notably in deep and linear subtypes, extracutaneous involvement, including loss of range of motion and joint deformity can occur [6]. The prognosis is generally favorable, with most patients seeing improvement in six to 12 months [7]. However, if morphea is not detected in an earlier phase, there is a risk of subcutaneous involvement leading to permanent disfigurement [1,7,8]. Furthermore, the risk of relapses requires long-term monitoring, especially in severe cases [9]. Detailed physical exams are appropriate, and serial imaging can be considered in these patients to monitor nervous system involvement. Takahashi et al. provide a graphic of the typical arrangement of skin lesions [10]. Several patterns of distribution are shown in Figure 1.

In the head, morphea invasion of deeper tissues is shown to cause neurological signs such as white matter lesions, cortical atrophy, focal brain abnormalities on MRI, as well as subclinical EEG changes. These can lead to subtle neurocognitive or neuropsychological deficits and, in rare cases, can cause seizures [11]. Morphea can present with neurosensations of burning, tingling, and muscle cramps when affecting the neck [12]. The risks of such manifestations are highest in patients with the linear subtype of morphea affecting the neck, and more specifically, with lesions affecting the cervical spine area [13,14]. Early screening of suspicious skin lesions affecting the skin over the cervical spine is important for preventing delayed treatment and subsequent subcutaneous manifestations [15]. While it has been generally characterized, the link between morphea in the cervical spine and the subsequent development of neurological manifestations has not been investigated thoroughly. As such, we aim to elucidate the implications of morphea in the cervical spine in this review.

Overview of morphea and neurologic involvement

Morphea classification is challenging due to its varying phenotypes. Laxer and Zulian published the Padua criteria, the most common classification in the present literature. The Padua criteria differentiate the following categories: circumscribed, linear, generalized, pansclerotic, and mixed subtypes. The most common subtype of morphea is circumscribed, presenting as plaques that can be broken down into superficial or deep forms. Linear morphea is differentiated into a limb/trunk variant and a head variant, including “en coup de sabre” (ECDS) and Parry-Romberg Syndrome (PRS) based on lesion morphology [1,8]. Linear morphea involves linear bands of sclerosis on the trunk, limbs, or head. Generalized morphea involves multiple plaques affecting more than two anatomical sites. Pansclerotic morphea is characterized by the involvement of skin and subcutaneous tissues, often with deep involvement. Finally, mixed morphea involves two or more of the previously mentioned subtypes [4,9]. Clinical presentation varies based on each phenotype, but skin lesions typically present as erythema, warmth, violaceous color, and varying degrees of induration [16]. 

While extracutaneous manifestations (neurologic, musculoskeletal, and ophthalmologic) are common in patients with morphea, linear morphea of the head (en coup de sabre) is well-known to be associated with neurological or ophthalmologic manifestations. The en coup de sabre type of morphea is described as a linear lesion of tissue across the head or the superior portion of the neck, and these lesions can cause neurologic manifestations [14]. Severity of the disease has not been reported to predict the extent of neurological involvement [17]. The most common neurological symptoms documented in the literature include non-migraine headaches, migraine headaches, and facial pain [14,18]. However, many patients can exhibit subclinical findings on physical exam, including cranial nerve abnormalities, abnormal deep tendon reflexes, and sensorimotor deficits in the extremities. Neurological symptoms are not correlated with abnormal neuroimaging findings, as imaging abnormalities may be present without clinical symptoms [17].

MRI can be useful in determining the depth of tissue involvement and progression of the disease [8,18]. However, the relationship between MRI findings and neurological symptoms has not been fully characterized. Fan et al. found that 50% of patients with abnormal imaging findings had no central nervous system (CNS) symptoms, and 43.3% of MRIs revealed underlying tissue damage that was not evident on physical exam [19]. This suggests that MRI imaging should be considered early in the evaluation of patients with a differential diagnosis, including morphea, ideally completed before treatment initiation to ensure a thorough assessment and timely treatment intervention. When diagnosing morphea, physicians should also keep a critical eye for subclinical symptoms, such as burning, itching, or muscle cramps [12]. These symptoms may be dismissed by patients; however, these subtle symptoms could be indicative of chronic, invasive morphea. If this invasive morphea went undiagnosed, there could be deep tissue changes and a higher likelihood of more significant disease.

## Review

Clinical presentation of morphea patients

There are several different types of morphea, and symptoms vary widely when this disease affects the cervical spine. Morphea can present with cutaneous symptoms, including skin dyspigmentation, erythema, dermal and subcutaneous atrophy, as well as destruction of deeper tissues [20]. Additionally, decreased range of motion or loss of strength of the musculature of the cervical spine can occur due to the excess tissue deposits in patients with cervical morphea. One case study reported that morphea can lead to the obstruction of lymphatic vessels [21]. However, some patients may not show symptoms for months, as was the case with a patient with keloidal morphea [15]. Keloid morphea is rare and presents as a firm, cord-like plaque with randomly entwined collagen bundles [15]. Because of the great variability in symptoms, careful physical exams and diagnostic imaging should be completed to ensure early detection and limit the progression of the disease. 

Some types of morphea present with neurologic and musculoskeletal signs and symptoms. Subtle symptom changes may precede changes seen on imaging. One study examining extracutaneous symptoms related to linear scleroderma identified that 47.2% of patients experienced articular symptoms and 17.1% experienced neurologic symptoms [22]. While there may be obvious general focal deficits in some cases of morphea, there can be more subtle symptoms felt by patients as well. Multiple mechanisms of these neurosensory symptoms in morphea have been suggested: autoimmune dysregulation leading to inflammation of nerves, or abundant collagen growth impinging on vasculature and causing nerve dysfunction [12]. These symptoms could easily be missed by patients and physicians when conducting a physical exam on morphea, so it is important for providers to be thorough, inquiring about subtle symptoms and employing diagnostic imaging when exams and symptoms are ambiguous. These minute-seeming details could be key in identifying and preventing the invasion of morphea into subcutaneous tissues. 

There is a range of treatment options for morphea depending on the extent of tissue involvement. More conservative measures include topical steroids, but treatments for systemic involvement include immunosuppressive agents [23]. For superficial treatment, topical corticosteroids or tacrolimus 0.1% are viable options, whereas for more progressive lesions that are widespread, methotrexate is the first-line medication [8]. In patients with joint contractures or who want cosmetic correction, surgery is the intervention of choice [24]. The tightening of the skin around the neck in morphea patients can cause underlying damage that requires surgery to correct. An article in the Journal of the American Society of Plastic Surgeonsstates that the main goal of surgery in these patients is recovery of soft tissue volume [24]. It is mentioned in the article that all surgeries were performed on facial structures, but similar goals of patient care apply for morphea involvement over the cervical spine. Depending on the depth and invasion of the disease, physicians across the domains of dermatology, rheumatology, orthopedic surgery, and plastic surgery may be needed to provide care to these patients.

Cervical spine involvement in morphea

The progression of morphea begins with inflammation, followed by subsequent sclerosis. In the early stages, thick collagen bundles form within the reticular dermis, and dense inflammatory infiltrates composed of lymphocytes, eosinophils, plasma cells, and macrophages migrate between these bundles. The epidermis is typically normal or has atrophied at this stage. Subsequently, in the fibrotic stage of morphea, there is less inflammation and instead thickened blood vessel walls with compact, eosinophilic collagen bundles. Here, the collagen may replace the underlying subcutaneous tissue, leading to a loss of soft tissue volume [2]. 

Though the atrophy in the late stage is poorly understood, morphea is capable of affecting the subcutaneous tissue and underlying fascia, muscles, and bones as well. The disease is classified based on the extent and depth of fibrosis, and musculo-articular manifestations, including myositis, fasciitis, and arthritis, leading to disfigurement and disability [2]. The fascial layers of the neck include the superficial cervical fascia (otherwise known as the subcutaneous tissue), the fibroadipose layer deep to the skin, and the deep cervical fascia. The latter layer includes the prevertebral fascia, which encloses the spine and the musculature surrounding the spine [25]. This may mark a pathway through which morphea lesions travel to reach the spinal column.

Musculoskeletal morphea primarily presents in the extremities, though cases of cervical spine involvement have also been observed. For example, a review of prospective cohort registries by Chen et al. found musculoskeletal manifestations in 4.9% and 2.8% of adults and children with morphea, respectively, and in another study, 11.5% of linear scleroderma patients with linear scleroderma presented with arthralgia [6,26]. These patients were treated with either medication or continued monitoring of clinical symptoms [26]. The medications used in the treatment groups included topical steroids, calcitrol, hydroxychloroquine, oral steroids, and methotrexate. Of note, in a study analyzing musculoskeletal involvement in the limbs of pediatric patients with morphea, 14% required surgical procedures [27]. It is recommended that surgical intervention only be considered during the inactive stage of morphea with at least six months of disease quiescence, as this will mitigate risk for reactivations [28,29]. There are several documented studies on facial involvement in morphea. However, interventions for these patients include fat grafting, hyaluronic acid filler injection, or surgical resection of the sclerotic tissue [30]. Morphea patients with deeper tissue involvement may require an orthopedic surgeon to restore muscle, tendon, and joint anatomy and physiology.

As for the effects of morphea on the cervical spine, common complications include limited range of motion, joint contractures, and inflammatory arthropathy [6]. Additionally, a separate study by Burke et al. identified that long-standing scarring can lead to scoliosis, myositis, fasciitis, and arthritis. They found that skin changes almost exclusively on the concave curve of the back were associated with underlying tissue involvement. After finding the right thoracic scoliosis, Burke and colleagues performed spine fusion with appropriate correction of the deformity [31]. Lastly, a study by Papara et al. showed growth retardation and muscle atrophy in addition to the aforementioned symptoms [2]. They also linked morphea with antinuclear, anti-single-stranded DNA, and anti-histone antibodies, with the latter two associated with joint and muscle involvement as well. Toledano et al. found antinuclear antibodies were positive in 7.7%, anti-double-stranded DNA in 1.9%, and rheumatoid factor in 7.7% of the participants in the study, showing no correlation to a localized scleroderma diagnosis [26]. This was a study of 52 patients that used the Mayo Clinic Classification for skin lesions, which differed from Papara et al. [2], so this is a possible explanation for the contrasting correlations with these serologic markers.

As for scleroderma, calcific masses and osteolysis can be present in the cervical spine, damaging the laminae, pedicles, lateral masses, or facets [32]. These masses result in pathology primarily by compressing the spinal cord; secondarily, they can produce spondylolisthesis and vertebral canal stenosis. Additionally, radiculopathy, focal pain, and stiffness have been observed in the cervical spine of patients with scleroderma [33].

Morphea can be identified radiologically via MRI, ultrasonography, and elastography. MRI is particularly salient in morphea, with findings such as fasciitis and myositis that allow one to identify the depth of infiltration and assess overall disease activity. Fascial thickening and enhancement are commonly observed, as well as articular synovitis, especially in pansclerotic morphea. Additional symptoms that have been found are tenosynovitis, subcutaneous septal thickening, and muscle and bone marrow edema. Of note, muscle and bone marrow edema were observed in 40% and 20% of MRIs, respectively [16]. Induration has also been identified as a sign of activity in deeper tissues when erythema is not visible at the surface [16]. In a study analyzing linear morphea, muscle atrophy, bone remodeling deformity, abnormal bone marrow signals, and joint contracture have all been documented. Outside the cervical region, limb length discrepancy, intracranial calcifications, and white matter abnormalities are also common in patients with morphea [34].

Clinical implications

Significance of Subclinical Findings

Nearly half of patients with head-and-neck morphea demonstrate intracranial or intraspinal abnormalities on MRI, despite a lack of neurological complaints [2,17]. In longitudinal case reports, incidental imaging findings were noted, and seizures, migraine, or the development of focal deficits developed months to years after these imaging changes [35]. As such, this quiescent phase represents an opportunity for immunomodulatory therapies aimed at preventing parenchymal damage before it becomes irreversible. Furthermore, cervical involvement represents a high-risk clinical subset of patients with morphea because induration of the paraspinal musculature or deep fascia can tether the dural sac, potentially compromising vertebral artery flow. This impeded blood flow can mimic a stroke-like state [16]. Induration, fibrosis, and loss of tissue compliance may restrict segmental motion and, over time, contribute to malalignment and compensatory postural changes. Chen et al. suggest that loss of cervical lordosis or development of a straightened or kyphotic neck contour may occur in these patients, potentially as a consequence of chronic fascial tethering, muscle spasm, or pain-avoidant posturing [6]. Taken together, these seemingly benign cutaneous plaques of the neck should prompt clinicians to have suspicion beyond the skin and anticipate a potentially progressive neuro-axial course.

*Diagnostic Challenges and Role of Imaging*

Cutaneous activity scores such as the Localized Scleroderma Assessment Tool (LoSCAT) have limited ability to gauge deep-tissue or neurologic disease, often leaving clinicians uncertain about when to escalate therapy [16]. MRI continues to be the most sensitive modality for detecting early marrow, fascial, and CNS changes, with high-resolution T2 and post-contrast sequences delineating edema before clinical deficits arise [8,36]. Guidelines formed subsequent to consensus-based recommendations from the Single Hub and Access point for paediatric Rheumatology in Europe (SHARE) consortium strongly recommend baseline brain MRI for every patient with craniofacial or scalp lesions and a musculoskeletal MRI, especially when plaques cross major joints [37]. For cervical disease, the same principle should be extended: a cervical spine magnetic resonance imaging (C-SPINE) MRI (C1-T2) should be obtained whenever neck lesions overlie the midline or are associated with dysesthesias, radicular pain, or unexplained range-of-motion loss [38]. Since violaceous borders of skin may underestimate fascial penetration, imaging can also be repeated if there is rapid induration of the skin or new neurologic complaints begin. This can ensure that therapeutic decisions are driven by objective depth-of-involvement criteria rather than surface appearance alone.

Screening Recommendations and Management

**Figure 2:**
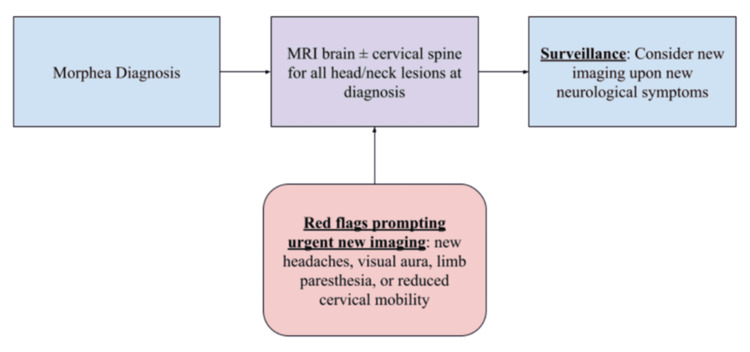
Recommended screening guidelines Guidelines adapted from recommendations in Knobler et al. (2023) [28].

Based on the *Journal of the European Academy of Dermatology and Venereology* 2024 consensus update, annual neurologic review and targeted imaging are advised for linear or deep subtypes; however, shorter intervals (six months) are recommended during active disease flares [39]. A practical algorithm is proposed in Figure 2.

Multidisciplinary care teams result in optimal care for patients, especially in the setting of complex disease with multi-system involvement [40]. In this scenario, patients would strongly benefit from a team that includes dermatologists to monitor cutaneous activity and systemic therapy, radiologists to interpret subtle neuro-axial changes, rheumatologists to monitor need and efficacy of pharmacologic therapies, and orthopedic or neurosurgical consults when structural instability or cord compression is suspected. Incorporating recommendations from this structured team into routine follow-up visits will not only streamline the decision-making process but will also shorten time-to-treatment escalation, a factor repeatedly associated with better functional outcomes [8,16]. Ultimately, adopting an early protocol for screening will transform morphea from a disease that is recognized only by late-stage complications into a more manageable condition that can be identified, anticipated, and controlled.

Research gaps and future directions

While the literature points to the magnitude of the neurologic manifestations of morphea, several important research gaps remain. Currently, there are well-known biomarkers for genetic predisposition of morphea, but there are no biomarkers to identify further neurologic risk stratification in morphea patients. In addition, cervical spine imaging provides unclear and unreliable markers of disease progression. The complex nature and unpredictable clinical course of this disease lead to barriers to delivering effective care. This warrants the need for further research.

Most studies available on morphea to date consist of case reports and small retrospective studies. Future research could look at prospective cohorts to provide data that will allow researchers to understand both causal relationships and long-term outcomes. There is also a lack of longitudinal studies in the literature. Further studies assessing the CNS and spinal progression of morphea in patients are crucial to understanding the disease process and thus treatment options.

## Conclusions

The current literature indicates that cervical involvement in morphea patients is a cause for concern due to the potential for deeper tissue infiltration. This infiltrative growth could affect the neurologic, musculoskeletal, or circulatory systems. Periodic monitoring with MRI and careful physical exams is the most effective way to monitor the progression of the disease. A team of clinicians is needed to evaluate and treat patients with morphea in the cervical spine. Case studies show that some patients may need the expertise of dermatologists, rheumatologists, and plastic and orthopedic surgeons to address the multi-system effects of morphea.

The subclinical findings are useful to help identify morphea at an early stage. In turn, treatment can be initiated sooner, leading to better outcomes for the patient. Morphea is generally self-limiting; however, once recognized, adequate medical care is essential to manage the patient’s symptoms. Early detection of the subtle neurologic signs provides physicians with the opportunity to limit further complications in the disease process. Because it is painless, it can be difficult to initially diagnose if the patient does not pay close attention to skin discoloration or hardening. The patient may not recall occasional numbness and tingling, but careful history taking is vital for the clinician to treat these patients. There can be serious complications that emphasize the need for early detection of morphea, especially in the cervical spine. Timely identification of these subclinical symptoms is important to gauge the depth of invasion of morphea.
